# Paraneoplastic Neurologic Disorders

**DOI:** 10.1007/s11910-023-01250-w

**Published:** 2023-02-13

**Authors:** Michael Gilligan, Christopher McGuigan, Andrew McKeon

**Affiliations:** 1 Department of Laboratory Medicine and Pathology, College of Medicine, Mayo Clinic, Rochester, MN, USA; 2 Department of Neurology, St Vincent’s University Hospital, Dublin, Ireland; 3 Department of Neurology, College of Medicine, Mayo Clinic, 200 1st ST SW, Rochester, MN 55905, USA

**Keywords:** Paraneoplastic neurologic syndromes, Autoimmune neurology, Diagnostic criteria, Immune checkpoint inhibitors

## Abstract

**Purpose of Review:**

To provide an overview and highlight recent updates in the field of paraneoplastic neurologic disorders.

**Recent Findings:**

The prevalence of paraneoplastic neurologic disorders is greater than previously reported and the incidence has been rising over time, due to improved recognition in the era of antibody biomarkers. Updated diagnostic criteria that are broadly inclusive and also contain diagnostic risk for clinical presentations (high and intermediate) and diagnostic antibodies (high, intermediate, and low) have replaced the original 2004 criteria. Antibody biomarkers continue to be characterized (e.g., KLHL-11 associated with seminoma in men with brainstem encephalitis). Some paraneoplastic antibodies also provide insight into likely immunotherapy response and prognosis. The rise of immune checkpoint inhibitors as cancer therapeutics has been associated with newly observed immune-mediated adverse effects including paraneoplastic neurological disorders. The therapeutic approach to paraneoplastic neurologic disorders is centered around cancer care and trials of immune therapy.

**Summary:**

The field of paraneoplastic neurologic disorders continues to be advanced by the identification of novel antibody biomarkers which have diagnostic utility, and give insight into likely treatment responses and outcomes.

## Introduction

Paraneoplastic neurologic disorders are heterogeneous autoimmune diseases occurring in the context of a non-nervous system cancer (solid organ or hematologic). They can arise as the clinical presentation for a previously undiagnosed cancer (e.g., a woman who presents with ataxia in whom ovarian adenocarcinoma is subsequently detected). Paraneoplastic neurological disorders can also arise during treatment for a recently diagnosed cancer, or can be the clinical presentation alerting the treating providers to a cancer relapse. These disorders can target any part of the neuraxis, rostrocaudally, from cerebral cortex to neuromuscular junction, though certain classical syndromes, now known as “high risk phenotypes,” have the highest risk for accompanying cancer (Table 1)[1••.

The diagnosis of a paraneoplastic neurologic disorder is usually supported by the detection of one or more neural antigen-directed IgG autoantibodies. These biomarkers serve to alert the clinician to the probability of malignancy as well as anatomical site and histologic type. While all these are biomarkers of various autoimmune neurological disease, the significance of each for a paraneoplastic diagnosis varies (Table 2 and Table 3). Some well-established biomarkers, such as antineuronal nuclear antibody type 1 (ANNA-1 [anti-Hu]) which has a > 70% positive predictive value for small cell (lung usually) carcinoma (SCLC) or other neuroendocrine lineage carcinomas, are considered high-risk [2]. More recently described biomarkers are considered either intermediate risk (with 30–70% risk for cancer, such as α-amino-3-hydroxy-5-methyl-4-isoxazolepropionic [AMPA]-receptor [R] antibody) or low risk (with 30% cancer risk, such as glial fibrillary acidic protein [GFAP]-IgG) [3, 4•]. Although novel antibody discoveries have increased diagnostic sensitivity, antibody negativity excludes neither a paraneoplastic cause nor cancer diagnosis [5••. Antigenic target type (intracellular versus cell surface) may also be informative for predicting treatment response and prognosis [6, 7]. Herein, we provide an update on paraneoplastic neurological disorders focusing on presenting phenotypes, antibody diagnostics, clinical assessment, and management.

## Epidemiology

Since early post-mortem studies first postulated a link between limbic encephalitis and carcinoma, appreciation of the prevalence of paraneoplastic neurologic disorders has grown [8]. Initially thought to affect only 0.01% of patients with cancer, it has been demonstrated more recently that paraneoplastic disorders occur in 1 in 300 patients with cancer [5••, 9]. Overall, they are most commonly associated with lung, breast, and ovarian carcinomas but specific syndromes carry their own particular cancer associations [5••. SCLC, with its inherent diverse neural antigen repertoire, carries the greatest risk for paraneoplastic neurologic disorders, occurring in approximately 10% of patients [10]. The prevalence of paraneoplastic neurologic disorders is 5.4 per 100,000 in the USA but increases to 11 per 100,000 for those > 60 years [11•]. The incidence rate across recent epidemiologic studies ranges from 0.4 to 1 per 100,000 person years [5••, 11•, 12]. Furthermore, the incidence of paraneoplastic neurologic disorders has increased over time, a trend consistent across multiple epidemiological studies [5••, 11•, 12].

In Northeastern Italy, the incidence of paraneoplastic neurologic disorders has almost doubled from 2009–2011 (0.62/100,000 person years) to 2015–2017 (1.22/100,000 person years) [5••]. A similar phenomenon was noted in Olmsted county Minnesota with incidence doubling from 1987–2002 (0.4/100,000 person years) to 2003–2018 (0.8/100,000 person years) [11•]. A similar trend was observed for autoimmune encephalitis which has trebled in incidence over 20 years, and is now more prevalent than infectious encephalitis [13].

These observations may be attributable to improved awareness among clinicians, increased detection through paraneoplastic autoantibody tests and profiles, and the advent of autoimmune complications of checkpoint inhibitor therapies for cancer [14, 15].

## Pathophysiology

Paraneoplastic neurologic disorders arise in the context of an immune response generated against antigens expressed on tumor cells which are also expressed natively in the host nervous system [16]. Antigens released following tumor cell apoptosis are presented by antigen-presenting cells to helper T cells in peripheral lymph nodes. CD4 + helper T cells subsequently activate antigen-specific B cells into to antibody-producing plasma cells. Disease mechanisms differ between disorders associated with antibodies to cell-surface antigens and those associated with antibodies directed against intracellular antigens. Antibodies against intracellular antigens and are not directly pathogenic but instead represent biomarkers of cytotoxic T cell-mediated cellular injury. This is supported by neuropathological studies which have revealed CD8 + T cell infiltration of neural tissues of patients with antibodies to intracellular antigens [7]. In contrast, antibodies directed against cell-surface antigens bind in vivo and in many instances pathogenic mechanisms have been characterized. For example, antibodies against n-methyl-d-aspartate (NMDA)-R, AMPA-R, and gamma amino butyric acid (GABA)_A_-Rs lead to neuronal dysfunction through a process of receptor cross-linking and internalization leading to reduced cell-surface receptor density [17]. GABA_B_-R antibodies, on the other hand, impair receptor function directly without causing receptor internalization [18]. LGI1 antibodies affect the protein–protein interaction with its receptor ADAM22, whereas aquaporin 4 antibodies mediate antigen internalization and complement-induced cytotoxicity [19–21].

## Clinical Presentations

### General Principles and Criteria

Neurological disorders are subacute in onset (over 6–12 weeks), and rapidly progressive. As per the updated diagnostic criteria, these disorders can be high-risk phenotypes (previously known as classic), and intermediate-risk phenotypes (Table 1). High-risk phenotypic presentations include encephalomyelitis, limbic encephalitis, rapidly progressive cerebellar syndrome, opsoclonus-myoclonus syndrome, sensory neuronopathy, enteric neuropathy, and Lambert-Eaton myasthenic syndrome. Intermediate-risk phenotypes include non-limbic encephalitides (such as anti-NMDA-receptor encephalitis), brainstem encephalitis, Morvan syndrome, isolated myelopathy, stiff-person spectrum disorders, and paraneoplastic polyradiculoneuropathies [1••. Other neurological disorders (e.g., paraneoplastic chorea, isolated myoclonus) or multifocal disorders (e.g., chorea with polyradiculoneuropathy) may occur in a paraneoplastic context [22, 23]. Thus, it is advisable to have some index of suspicion for a paraneoplastic disorder in patients presenting with a subacute neurological illness where an alternative cause is not immediately obvious.

In cases where an antibody is detected, it is possible to stratify the likelihood of cancer according to the specific antibody. Antibodies are termed “high risk” when the likelihood of cancer is > 70%, “intermediate risk” when there is a 30–70% association with cancer, and “low risk” when the probability of cancer is < 30%, Tables 2 and 3 [1••]. The PNS-Care score allows possible, probable, and definite levels of paraneoplastic diagnostic certainty, based on the level of risk from the neurological phenotype, antibody detected, and cancer found [1••]. For example, a woman with cerebellar ataxia (high risk syndrome, = 3), PCA-1 (high risk antibody, = 3), and ovarian adenocarcinoma (cancer consistent with phenotype and antibody, = 4) would have a score of 10, and thus fulfill definite criteria (score ≥ 8 required).

Although paraneoplastic neurologic disorders often precede cancer detection, most cases occur in the context of an existing cancer diagnosis [5••]. The timing of cancer presentation may vary, depending on the antibody specificity. For example, most Purkinje cytoplasmic antibody type 1 (PCA-1, anti-Yo) paraneoplastic cerebellar degeneration cases manifest subsequent to cancer diagnosis, whereas the reverse is true in Kelch-like protein (KLHL)-11 autoimmunity [24–26, 27•. When the neurological disorder is encountered first, 92% of cancers are detected within 5 years of symptom onset [5••]. However, recent consensus guidelines note that in a majority of cases, cancer is identified within 2 years of the onset of symptoms [1••]. For example, the mean interval to cancer diagnosis is 6 months in KLHL-11 rhombencephalitis, 6.5 months in ANNA-1 encephalomyelitis, and 9 months in ANNA-2-(anti-Ri)-associated disorders [2, 27•, 28]. A list of intracellular and cell-surface antibodies along with their clinical and tumor associations are provided in Tables 2 and 3. Paraneoplastic neurologic disorder phenotypes are considered in rostrocaudal order below.

## Central Nervous System

### Limbic Encephalitis

Limbic encephalitis is considered a high-risk paraneoplastic phenotype. Patients with limbic encephalitis present with subacute onset and rapid progression over approximately 3 months with working memory deficits, seizures, or psychiatric symptoms. Those with typical limbic encephalitis have bilateral T2 signal abnormalities of limbic structures on MRI, and at least one of CSF pleocytosis or EEG with temporal lobe findings (slow waves, seizures, or both). Patients with a paraneoplastic antibody could meet diagnostic criteria without fulfilling all of those criteria (EEG, CSF white cell count, and MRI findings) [29]. Paraneoplastic limbic encephalitis occurs most commonly in association with ANNA-1 and CRMP-5-IgG. SCLC is the most frequent neoplasm [30]. In the experience of the authors, forme frustes of limbic encephalitis can also occur, mostly isolated limbic seizures without cognitive impairment, though usually this occurs in a non-paraneoplastic context such as LGI1 or GAD65 autoimmunity.[31].

### Encephalitis (Extra-limbic)

Extra-limbic encephalitis is considered intermediate risk for a paraneoplastic cause. The term “extra-limbic” refers to clinical, radiological, or EEG findings indicative of neocortical temporal or extra-temporal localization, including multifocal disorders. Examples include anti-NMDA-receptor encephalitis, gamma-aminobutyric acid-A receptor (GABA_A_-R) encephalitis, dipeptidyl-peptidase-like protein (DPPX) encephalitis, and autoimmune GFAP astrocytopathy. Clinical and radiological presentations are diverse (Table 2). Anti-NMDA-R encephalitis classically manifests with a psychiatric prodrome followed by seizures, movement disorders, autonomic instability, and coma. Normal MRI and CSF pleocytosis are typical and neoplasm (usually ovarian teratoma) occurs in 38% [32, 33]. GABA_A−_R encephalitis usually presents with a multifocal extra-limbic syndrome with seizures, with multifocal non-enhancing deep or subcortical white matter lesions sometimes in association with thymoma [34, 35]. DPPX encephalitis classically presents with a triad of gastrointestinal disturbance, CNS hyperexcitability (spasms, myoclonus), and encephalopathy, with normal MRI imaging, in association, rarely, with B cell neoplasia [36, 37]. Autoimmune GFAP astrocytopathy presents with various neuropsychiatric symptoms, meningeal symptoms (blurred vision, headache) and sometimes tremor, or myelopathic sensory and autonomic findings, accompanied by CSF pleocytosis. MRI imaging typically includes diverse patterns of post-gadolinium enhancement (classically following the path of deep white matter glial cells running perpendicular to the corpus callosum). Approximately one-third have accompanying neoplasms of diverse histologies [38, 39].

### Encephalomyelitis

Patients with encephalomyelitis have a multifocal neurological disorder with symptoms referable to both spinal cord and cerebral cortex, but which may also include symptoms localizing to peripheral nerve, nerve root, or dorsal root ganglia [40]. Terms such as encephalomyeloneuritis or encephalomyeloradiculitis are also employed to more precisely define the clinical phenotype. Paraneoplastic encephalomyelitis is considered a high-risk phenotype and typically has an association with SCLC and one or more of ANNA-1, CRMP-5, and amphiphysin antibodies [2, 41, 42]. Among more recently described syndromes, autoimmune GFAP astrocytopathy (a low risk antibody) may present with encephalomyelitis, though usually with distinctive CSF pleocytosis and inflammatory-appearing T2-or T1-post gadolinium findings within cerebral white matter and central spinal cord. Associated cancers include adenocarcinomas or ovarian teratoma [38]. In myelin oligodendrocyte glycoprotein antibody disease, a report of encephalomyelitis in the context of a MOG protein-expressing teratoma suggests the potential to rarely occur as a paraneoplastic phenomenon [43].

### Brainstem Syndromes

Paraneoplastic rhombencephalitis presents with symptoms localizing to the brainstem and cerebellar connections including gait disturbance, postural instability, oscillopsia, vertigo, diplopia, dysarthria, dysphagia, sleep disorders (such as sleep disordered breathing, stridor, dream enactment behavior), and cranial neuropathies [44]. Supranuclear gaze palsies can resemble those of progressive supranuclear palsy [45]. Specific signs suggestive of certain antibody specificities include jaw dystonia and ANNA-2 (in the setting of breast adenocarcinoma) and vestibulocochlear symptoms (vertigo, tinnitus and hearing loss) in men with KLH-11 antibody (seminoma in 70%) [26, 27•, 28]. Listeria rhombencephalitis is an important subacute-onset differential diagnostic consideration [46].

### Rapidly Progressive Cerebellar Ataxia

This high-risk phenotype presents with gait, speech, and dysmetric limb findings (a pancerebellar disorder) with significant disability accruing over 12 weeks. More rapid and slowly progressive forms have also been described [47]. For this phenotype, a diverse range of antibodies (paraneoplastic and non-paraneoplastic) could be considered, starting with those more common with higher cancer risk (such as PCA-1 [ovarian and breast adenocarcinoma associated], PCA-Tr and metabotropic glutamate receptor [mGluR]-1, which are both lymphoma associated), while research-based testing could be considered for the less common and infrequently cancer-associated analytes (such as inositol triphosphate receptor [ITPR]-1 and septin-7 antibodies) [25, 48–51]. Distinct clinical features may aid in the diagnosis of certain ataxias such as loss of taste sensation in mGluR1 ataxia and episodic ataxia which can arise in the context of contactin-associated protein-like 2 (CASPR2) autoimmunity (sometimes accompanying thymoma) [52, 53].

### Opsoclonus-Myoclonus Syndrome

Opsoclonus-myoclonus syndrome (OMS) is subjectively characterized by generalized tremulousness and oscillopsia, and objectively by arrhythmic, multidirectional conjugate saccadic eye movements (opsoclonus) accompanied by limb and trunk myoclonus. Other clinical features may include ataxia, encephalopathy, and sleep disturbance [54]. In children, opsoclonus-myoclonus is strongly associated with neuroblastoma [55]. Among adults, OMS mostly arises as an idiopathic autoimmune phenomenon. Paraneoplastic antibodies encountered include ANNA-2 antibodies (less commonly ANNA-1). Breast adenocarcinoma and SCLC are the more frequently reported oncological accompaniments [54, 56]. OMS accompanying encephalopathy sometimes occurs in anti-NMDA-R encephalitis.

### Myelopathy

Isolated paraneoplastic myelopathy manifests with subacute or insidiously progressive spinal cord signs such as motor weakness, bowel, or bladder disturbance. It can also present with tract-specific signs such as a dorsal column syndrome. Associated antibody specificities include CRMP-5, amphiphysin, ANNA-1, and neuronal intermediate filaments (particularly neurofilament light chain) [57•, 58]. Small cell carcinoma and breast adenocarcinoma are the typical neoplastic associations [58]. Although more commonly an idiopathic autoimmune phenomenon, a paraneoplastic myelitis can occur in the setting of neuromyelitis optica spectrum disorder (NMOSD) mediated by aquaporin-4 (AQP4) antibodies. Neoplastic accompaniments include thymoma, and breast and lung carcinomas [59]. Older age of onset, especially in men, is a risk factor for paraneoplastic NMOSD [60].

### Stiff Person Syndrome

Paraneoplastic stiff person syndrome (SPS) accounts for just 1–2% of all SPS cases [61]. Stiff person syndrome arises usually as an idiopathic autoimmune phenomenon associated with glutamic acid decarboxylase (GAD65) or glycine receptor antibodies [61]. The classical SPS clinical presentation includes muscle rigidity and spasms, symmetrically involving the trunk and proximal lower limbs. Symptoms are exacerbated by emotional distress or startle. Partial forms affecting one limb or the trunk in isolation have also been described. In rare cases of SPS, usually among older male patients, GAD65 antibodies may occur in association with lung, breast, or thymic neoplasms [62]. In the setting of glycine receptor autoimmunity, the progressive encephalomyelitis with rigidity and myoclonus (PERM) phenotype is most common [63]. This represents SPS findings in the context of a widespread encephalomyelitis. Though usually idiopathic in etiology, lymphoma and thymoma are among reported neoplastic accompaniments [64].

## Peripheral Nervous System, Neuromuscular Junction, and Muscle

### Polyradiculopathy

Paraneoplastic polyradiculopathy or polyradiculoneuropathy occurs in isolation or as part of a multifocal encephalomy-eloneuropathic disorder. CRMP-5 polyradiculoneuropathy usually presents with a painful, asymmetric axonal neuropathy. Amphiphysin autoimmunity is associated with a symmetric axonal neuropathy [66–68]. Microtubule-associated protein 1B (MAP1B) autoimmunity more commonly presents with a painless polyradiculoneuropathy [69•]. The most common oncological association for MAP1B and CRMP-5 IgGs is small cell carcinoma. Adenocarcinoma of breast or SCLC are the usual oncologic accompaniments of amphiphysin-IgG [67, 68, 69•].

### Subacute Sensory Neuronopathy

Subacute sensory neuronopathy is a high-risk paraneoplastic phenotype in which the disorder localizes to the dorsal root ganglion. Patients develop sensory loss subacutely over weeks or months, initially affecting vibration and proprioception, followed by involvement of the other sensory modalities. The clinical picture is one of profound sensory ataxia often with accompanying pseudoathethosis on clinical examination [70]. The presence of ANNA-1 and accompanying SCLC is prototypic, but subacute sensory neuronopathy can also arise in the setting of CRMP-5, amphiphysin, and MAP1B antibodies [67, 68, 69•, 71].

### Autonomic Neuropathy

Chronic gastric pseudo-obstruction represents a focal form of autoimmune autonomic neuropathy. This disorder presents with abdominal distension, cramping, nausea, vomiting, and weight loss not explained by mechanical obstruction. Other or additional gastroenterologic localizations may be encountered, namely small-bowel pseudo-obstruction or large-bowel obstipation. One or more other features of dysautonomia may accompany the gastroenterologic complaints (orthostatic hypotension, mydriasis, heat intolerance due to anhidrosis, erectile dysfunction, and urinary retention) [72, 73]. Serological findings include ANNA-1, in the setting of small cell carcinoma, or (occasionally in the authors’ experience) thymoma. Autoimmune autonomic ganglionopathy with ganglionic nicotinic acetylcholine receptor (α3-AChR) antibody detected is occasionally paraneoplastic in etiology, associated with diverse cancer types, including small cell carcinoma and thymoma [73, 74]. When autonomic neuropathy and sensorimotor neuropathy co-exist, the typical oncological accompaniment is SCLC [74].

### Neuromuscular Junction Disorders

Lambert Eaton myasthenic syndrome (LEMS) is a high-risk paraneoplastic phenotype accompanied by small cell carcinoma in 50% generally, with an even higher risk in smokers older than 50 with weight loss [75]. Though P/Q-type calcium channel antibody is a general biomarker for LEMS, coexisting antiglial/neuronal nuclear antibody (AGNA, or Sry-like high-mobility group box protein 1 [SOX-1]-IgG) or ANNA-1 positivity is more predictive of small cell carcinoma in those cases [76]. In a study assessing for risk for SCLC in LEMS patients, age at onset, smoking behavior, weight loss, Karnofsky performance status, bulbar involvement, male sexual impotence, and the presence of SOX-1 antibody were independent predictors [77]. A DELTA-P score was derived allocating 1 point for the presence of each of the following items at or within 3 months from onset: age at onset ≥ 50 years, smoking at diagnosis, weight loss ≥ 5%, bulbar involvement, erectile dysfunction, and Karnofsky performance status lower than 70. A DELTA-P score of 0 or 1 corresponded to a < 3% chance of SCLC, whereas a score of ≥ 4 or more had a positive predictive value for SCLC of > 90% [77]. Myasthenia gravis is associated with thymoma in 10–15% of cases [78].

### Neuromyotonia

Peripheral nerve excitability, or neuromyotonia, is characterized by stiffness, muscle cramps, fasciculations, and/or myokymia. Dysautonomia including hypohidrosis, orthostatic hypotension, and gastric dysmotility may co-occur in patients with LGI1 or CASPR2 antibodies [79]. Morvan’s syndrome is the co-occurrence of neuromyotonia with central nervous system dysfunction such as encephalopathy, seizures, and insomnia (termed “agrypnia excitata”). Morvan’s syndrome carries a 50% risk of thymoma, usually accompanying CASPR2 antibodies [80, 81].

### Myopathy

Dermatomyositis is a form of immune-mediated myopathy which carries a 15% risk of malignancy [82]. It presents with proximal muscle weakness and cutaneous findings such as a periorbital heliotrope rash or violaceous papules over the dorsal aspect of the metacarpophalangeal joints (“Gottron papules”). Dermatomyositis is associated with a wide range of malignancies and the histologic type varies according to ethnicity and underlying population risk profile [83]. Certain antibodies such as nuclear matrix protein 2, transcription intermediary factor 1 gamma, and small ubiquitin-like modifier 1 activating enzyme subunit occur more frequently in paraneoplastic dermatomyositis [84]. Necrotizing autoimmune myopathy is characterized by painful, proximal muscle weakness and can occur as a paraneoplastic phenomenon in 10% of cases, some of whom are signal recognition particle (SRP) antibody positive. The most frequent cancer association is gastrointestinal adenocarcinoma [85].

## Diagnostic Evaluation

### Clinical Assessment

The neurological clinical assessment should pay particular attention to the trajectory of symptom onset and progression. In almost all circumstances, onset is subacute. Neurological exam should assist in localizing the disorder to one or more anatomic regions of the nervous system. Risk factors to consider when evaluating paraneoplastic neurologic disorders include a current or remote history of malignancy, smoking history, coexisting non-neurologic autoimmune disease history, and exposure to immune checkpoint inhibitors.

### Imaging

#### MRI

**Fig. 1:**
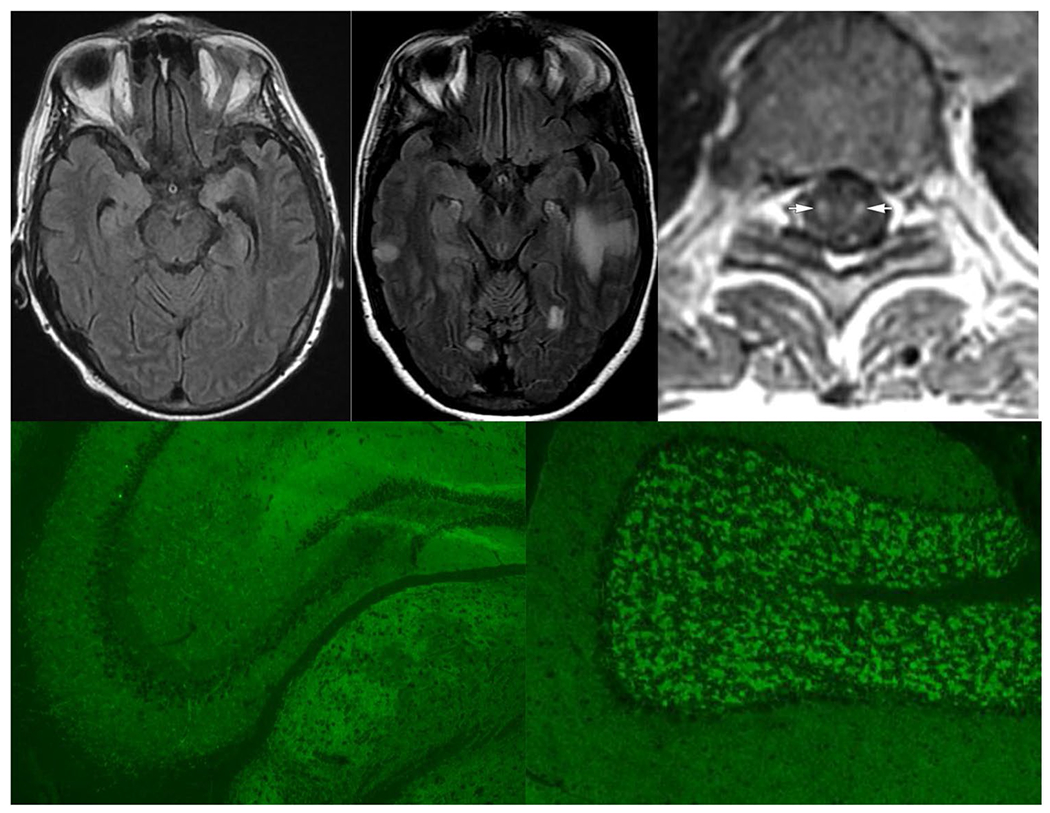
MRI and antibody test findings in paraneoplastic disorders. Top row, axial MRI images demonstrate (left, T2 FLAIR) bilateral mesial temporal hyperintensities in a patient with ANNA-1-associated paraneoplastic limbic encephalitis (middle, T2 FLAIR) multifocal extra-limbic hyperintensities in a patient with GABA-A receptor (R) encephalitis, and (right, T1 post-gadolinium) spinal cord tractopathies in a patient with seronegative renal cell carcinoma-associated paraneoplastic myelopathy. Bottom row, indirect immunofluorescence assay using mouse brain tissue as substrate reveals a typical synaptic pattern of IgG staining of hippocampus (left) and cerebellum (right) produced by CSF from a patient with GABA-A-R encephalitis. GABA-A-R specificity was confirmed by GABA-A-R alpha 1 subunit specific cell-based assay (not shown). Top right image from reference 58 reproduced with permission from American Academy of Neurology

In paraneoplastic limbic encephalitis, T2 hyperintensity of the mesial temporal lobes is typical, occasionally with contrast enhancement (Fig. 1) [86]. In extra-limbic paraneoplastic encephalitides, MRI may demonstrate extra-temporal lobar abnormalities that can resemble infectious or neoplastic processes [87]. Linear, radial enhancement extending from the lateral ventricles is a characteristic MRI imaging finding in GFAP astrocytopathy [38]. Distinctive radiological features also arise in GABA_A_-R encephalitis characterized by multi-lobar deep white matter and juxtacortical lesions on FLAIR imaging (without enhancement), Fig. 1 [88]. In many instances, MRI of the brain can be entirely normal, including most patients with anti-NMDA receptor encephalitis [32].

The hallmark radiographic feature of paraneoplastic myelitis is longitudinally extensive, gadolinium-enhancing T2 signal change selective for individual spinal tracts, sometimes with enhancement post-gadolinium (Fig. 1). This finding most commonly affects the dorsal and lateral columns. However, the MRI may be normal in half of all cases [66, 89].

Nerve root enhancement is a radiological feature present in one-third of patients with paraneoplastic myeloneuropathy [66]. In paraneoplastic myopathy, MRI can support the diagnosis, localize the affected muscles for a targeted biopsy, and in certain cases distinguish between myositis subtypes [90].

### Neurophysiology

Neurophysiological testing can aid in localizing a paraneoplastic neurologic disorder. The extreme delta brush pattern on EEG occurring in one-third of patients with anti-NMDA receptor encephalitis is associated with more severe illness [91]. Slow wave or epileptiform activity localized to the temporal lobe may aid reaching a diagnosis of limbic encephalitis [29]. Continuous motor-unit activity on EMG, agonist–antagonist co-contraction, exaggerated acoustic startle, and exteroceptive responses are characteristic of stiff person syndrome [92]. In neuromyotonia, nerve conduction studies reveal stimulus-induced after discharges and EMG characteristically demonstrates spontaneous firing of doublet or multiplet motor unit discharges indicative of peripheral nerve hyperexcitability [93]. Pre- and post-synaptic neuromuscular junction disorders can be distinguished by compound muscle action potential amplitude responses to repetitive nerve stimulation (increment in LEMS, decrement in myasthenia gravis) [94].

### Immunological testing

#### Antibody testing

Neural antibodies, which are of IgG class, belong to 2 broad groups, those mostly “high risk” and reactive with linear epitopes of nuclear (e.g., Hu), nucleolar (e.g., Ma2), or cytoplasmic (e.g., Yo) antigens, and those of “intermediate” or “low” risk mostly reactive with cell surface protein conformation-dependent extracellular epitopes, such as GluN1 subunit of NMDA receptor (Tables 2 and 3). Antibody testing should be performed in both serum and CSF in a laboratory with expertise diagnosing autoimmune neurologic disorders. Certain IgGs are more readily detected in serum (e.g., LGI1 antibody) and others are more readily and specifically detected in CSF (e.g., NMDA-R and GFAP antibodies) [38, 79, 95]. Tissue-based immunohistochemistry or immunofluorescence serves as a screening test for most antibodies; a high degree of interpretative experience is necessary to precisely identify these (Fig. 1). IgG specificities identified should be confirmed by protein-specific methods, such as western blot, ELISA or radioimmunoprecipitation assays for intracellular antigens, and transfected cell-based assays (observer-based or flow cytometry) for cell-surface antibodies. It is not recommended to rely on commercial line blots alone and positive results, where possible, should be correlated with tissue immunohistochemistry [96•]. In the authors’ experience, certain antibodies are not sensitively detected by tissue-based assays (e.g., LGI1, CASPR2, AQP4-IgG, and MOG-IgG), but are detectable sensitively and specifically by optimized cell-based assays, either by microscopy or by flow cytometry [97]. The absence of an antibody does not preclude the diagnosis of a paraneoplastic neurologic disorder. Where a high index of suspicion persists in seronegative cases, serum and CSF samples could also be referred to a laboratory with research expertise for novel antibody testing [1••].

#### CSF

Routine findings on CSF that are supportive of a paraneoplastic neurologic disorder include pleocytosis, the presence of CSF-exclusive oligoclonal bands or a raised CSF IgG index or synthesis rate. Cytology should be performed to exclude direct rather than remote effect of systemic cancer. CSF glucose levels are not abnormal in paraneoplastic neurologic disorders but can be reduced in metastatic cancer or infection [98]. Normal CSF parameters do not exclude a paraneoplastic diagnosis [99].

### Cancer Screening

Cancer testing may be quite selective, when guided by the antibody detected, or could be a broad search in seronegative patients or in those with an antibody with less specific cancer associations. CT imaging of chest, abdomen, and pelvis is usually pursued. This could be supplemented, as appropriate, with CT of the neck, ultrasound, or MRI of the gynecologic tract, ultrasound of testes, pelvic MRI, mammography, and gastroenterologic endoscopies. Fluorodeoxyglucose-PET-CT (orbits to thighs) is useful for detecting carcinoma or lymphoma in primary screening, or after normal CT imaging [100]. High-resolution CT or MRI is preferred for thymoma detection. Paraneoplastic antibodies may direct the search for cancer and related management decisions. For example, in a man with KLHL-11 or Ma2 antibodies, testicular seminoma or other germinoma would be suspected [27•, 44]. For patients with Ma2 antibodies, a compatible neurologic phenotype and testicular microcalcifications on ultrasound, orchiectomy would be considered for patients under 50 years [1••]. Similarly, in post-menopausal women with PCA-1, exploratory surgery or prophylactic hysterectomy and bilateral salpingo-oophorectomy might be considered even if imaging tests were negative [1••]. While improvements may be encountered in KLHL-11 patients (50%), they are almost always absent in PCA-1 patients (85%) [25, 26]. For patients with a high-risk antibody accompanied by a compatible neurologic syndrome without underlying cancer, it is recommended that tumor screening should be repeated every 4–6 months for 2 years. The same applies for intermediate-risk antibodies but only if accompanied by a high-risk phenotype (Table 1). For low-risk antibodies, cancer screening at the time of diagnosis is deemed sufficient; however, decisions should be guided by individual patient assessment, presence of risk factors, and clinical judgment [1••].

### Pathology

In the context of suspected autoimmune encephalitis, occasional patients meet the criteria for seronegative probable autoimmune encephalitis on the basis of inflammatory brain biopsy findings, and one other supportive finding (inflammatory CSF or MRI) [29]. In that setting, a search for cancer might be undertaken, though the overall risk for cancer in a seronegative non-limbic encephalitis is low [101]. In myositis, specific features on muscle biopsy aid in arriving at a diagnosis of dermatomyositis or immune-mediated necrotising myopathy [102]. Though not done routinely, a paraneoplastic diagnosis may be strengthened by detecting the cognate antigen for a low-risk antibody in the patient neoplasm by immunohistochemistry [1••].

## Immune Checkpoint Inhibitors

Immune checkpoint inhibitors (ICIs) are a class of monoclonal antibodies which inhibit the immune-checkpoints that exert a negative regulatory effect on the immune system [103]. Targets include cytotoxic T-lymphocyte antigen 4 (CTLA-4: ipilimumab), programmed cell death protein 1 (rembrolizumab, nivolumab), and programmed cell death ligand 1 (durvalumab) [104]. These therapies restore host antitumour immunity, promote tumor cell death, and have been associated with improved outcomes even in patients with advanced malignancy [105]. Novel toxicities termed immune-related adverse events (irAEs) have emerged in association with ICIs. Neurologic irAEs (nirAE) are thought to affect 1% of patients and include myositis, myasthenia gravis, and inflammatory neuropathies including Guillain-Barré syndrome [106]. This risk increases when anti-CTLA-4 is used in combination with anti-PD-1/anti-PD ICIs [107, 108]. Neuromuscular and peripheral nirAEs are more common than disorders with central nervous system involvement, and myasthenia gravis has the highest fatality rate among all nirAEs [109].

In 2019, Graus and colleagues defined three clinical scenarios where nirAEs can be considered to meet criteria for paraneoplastic neurologic disorders: (i) when the symptoms of the nirAE are identical to that of a classical paraneoplastic neurologic disorders, irrespective of the presence of paraneoplastic antibodies; (ii) any nirAE in association with the detection of paraneoplastic antibodies provided other causes are excluded; (iii) nirAEs in the presence of cell-surface protein or synaptic antibodies in the presence of a typical tumor [110••].

Paraneoplastic neurologic syndromes often respond poorly to systemic immunotherapy due to irreversible cytotoxic T cell–driven neuronal injury [16]. However, in cases associated with ICI use, withdrawal of the ICI in addition to treatment with one or more of corticosteroids, IVIg and plasma exchange can be beneficial [14]. Administration of corticosteroids in particular has been associated with favorable clinical outcomes, even in disorders that do not typically demonstrate a favorable response to steroids (e.g., Guillain-Barré syndrome) [14, 109]. Seronegative cases have been reported in the context of ICI use and therefore the absence of high or intermediate-risk antibodies does not exclude the diagnosis [111].

## Treatment

Treatment includes nervous system–directed immunotherapy, cancer-specific treatment, and symptomatic therapy.

### Immunotherapy

In general, disorders associated with cell-surface antibodies respond to immunotherapy better than other paraneoplastic disorders, whereas those associated with intracellular anti-neuronal antibodies respond less favorably [16]. Class 1 evidence for immunosuppressant therapy is lacking and treatment strategies are guided by antibody type (cell surface-directed versus intracellular), expert opinion, and small case series. Improvements may be modest in the case of disorders associated with antibodies to intracellular antigens and arresting further neurological decline is often the therapy goal. It is recommended that baseline neurological assessments be obtained and recorded prior to initiation of therapy to evaluate the degree of treatment response. A trial of immunotherapy typically consists of IV methylprednisolone 1 g and/or intravenous immunoglobulin (IVIg) 0.4 g/kg daily for 5 days and can occur during or after cancer treatment. This may be followed by weekly doses for 6–12 weeks, or a slow oral taper of prednisone. Plasmapheresis every second day for 5–7 treatments can also be trialed upfront or used in treatment-resistant cases. In syndromes associated with intracellular antigen-directed antibodies, neuronal injury is evidentially cytotoxic T cell–mediated and therefore cyclophosphamide can be used [112]. Rituximab has generally been considered a second-line agent in patients with disorders associated with cell-surface antibodies, though in anti-NMDA-R encephalitis it is frequently used early in the disease course of patients with severe neurological disease without waiting for outcomes of first-line therapy (steroids and IVIg or plasma exchange) [113].

Relapsing disease is uncommon, though it can occur in 20–30% of patients with certain intermediate or low-risk antibodies such as anti-NMDA-R encephalitis, autoimmune GFAP astrocytopathy, PERM, and LgI1 encephalitis [32, 38, 64, 79]. For those relapsing patients, maintenance immunotherapy considerations include intravenous rituximab or oral agents such as azathioprine, mycophenolate mofetil, or methotrexate. For cases requiring chronic therapy, the weaning of intravenous methylprednisolone or IVIg should occur in a cautious manner, extending the dosing interval gradually over 3–6 months, to mitigate against the risk of relapse. For paraneoplastic disorders in the setting of ICI use, the treatment involves withdrawal of the ICI and administration of corticosteroids (IV regimen as detailed above or oral prednisolone 60–80 mg for 1–2 weeks) [14]. There is no established consensus regarding the duration of chronic immunotherapy in paraneoplastic neurologic disorders.

### Cancer Treatment

The treatment of cancer, as guided by oncology and surgery, may coincide with stabilization or improvement of neurological symptoms. Some patients with anti-NMDA-R encephalitis have marked neurological improvements after ovarian teratoma resection [32]. However, because this response is variable, immune therapy is universally recommended in addition to teratoma removal [113]. In many cases, cancer treatment may at best result in stabilization of the neurological disorder such as in PCA-1 cerebellar ataxia [25].

### Symptomatic Therapy

Anti-seizure medications should be trialed for symptomatic seizures but immunotherapy is characteristically more effective for seizure control [114]. Benzodiazepines (usually diazepam) are used for SPS [61]. Other movement disorders may respond to symptomatic therapy such as parkinsonism (levodopa), myoclonus (benzodiazepines), or dystonia (trihexyphenidyl or botulinum toxin). Carbamazepine or phenytoin are employed to manage muscle cramps and stiffness in paraneoplastic neuromyotonia [115]. Pyridostigmine is used in gastrointestinal pseudo-obstruction, myasthenia gravis, and LEMS (with 3,4 diaminopyridine for the latter) [115–117]. Neuropathic pain medications such as gabapentin, pregabalin, and tricyclic anti-depressants are employed in paraneoplastic neuropathy, but cancer treatment remains the optimal means of stabilizing symptoms [117].

## Conclusions

The field of paraneoplastic neurologic disorders has been advanced through updated diagnostic criteria aiding classification. The prevalence of these disorders is greater than previously reported. With increased use of immune-checkpoint inhibitors, it is possible that the incidence of paraneoplastic neurologic disorders will continue to rise. IgG biomarkers have diagnostic, therapeutic, and prognostic utility and can guide management decisions. Many paraneoplastic neurologic disorders remain seronegative.

## Figures and Tables

**Table 1:** Paraneoplastic phenotypes: high risk, intermediate risk, other

**High-risk phenotypes** Limbic encephalitis Encephalomyelitis Rapidly progressive cerebellar syndrome Subacute sensory neuronopathy Gastro-intestinal pseudo-obstruction Lambert Eaton myasthenic syndrome Opsoclonus myoclonus Dermatomyositis
**Intermediate-risk phenotypes** Encephalitis (extra-limbic) Brainstem encephalitis Morvan syndrome Isolated myelopathy Stiff person syndrome Polyradiculopathy
**Other** Acquired neuromyotonia (Isaac’s Syndrome) Autoimmune autonomic neuropathy Myasthenia gravis Necrotizing autoimmune myopathy

**Table 2:** Paraneoplastic antibodies (high or intermediate risk and common): neurological and oncological accompaniments

Antibody (alternative name)	Anitgen location	Risk	Clinical Manifestations	Associated neoplasm type
ANNA-1 (Hu) [2]	Intracellular	High	Sensory neuronopathy, ataxia, limbic encephalitis, opsoclonus myoclonus, autonomic neuropathy, gastrointestinal pseudoobstruction	SCLC, thymoma, neuroendocrine, neuroblastoma
ANNA-2 (Ri) [28, 118]	Intracellular	High	Ataxia, opsoclonus myoclonus, dystonia, and parkinsonism	Breast adeno, SCLC
PCA-1 (Yo) [25]	Intracellular	High	Ataxia, occasionally myelopathy, or peripheral neuropathy	Ovary, breast adeno
MAP1B (PCA-2) [69•]	Intracellular	High	Ataxia, encephalomyelitis, sensorimotor neuropathy	Lung (SCLC and NCSLC), breast adeno
ANNA 3 (DACH1) [119]	Intracellular	High	Ataxia, sensorimotor neuropathy, cognitive disorders, dysautonomia	SCLC
Ma2 (with or without Ma1) [120]	Intracellular	High	Limbic encephalitis, brainstem encephalitis, narcolepsy/cataplexy	Testicular germinoma in men; Lung, testicular, GI, breast adenos, or NHL in either sex
Amphiphysin [42, 68]	Intracellular	High	Stiff person syndrome, encephalitis, myelopathy, cerebellar syndrome	SCLC, breast adeno
AGNA (SOX1) [121]	Intracellular	High	Ataxia, encephalitis, LEMS	SCLC
Kelch-like Protein 11[26, 27•]	Intracellular	High	Ataxia, brainstem encephalitis, diplopia, hearing loss, and vertigo	Testicular (seminoma), teratoma
CRMP5 (CV2) [67, 122]	Intracellular	High	Encephalomyelitis, sensory neuronopathy, ataxia, LEMS, uveal/retinal involvement,	SCLC, thymoma
mGluR1 [50]	Cell surface	Low	Ataxia	Hodgkin lymphoma, cutaneous T cell lymphoma
PCA-Tr (DNER) [49]	Cell surface	High	Ataxia	Hodgkin lymphoma
AMPAR [3]	Cell surface	Intermediate	Limbic encephalitis	SCLC, malignant thymoma, breast, ovarian adenos
GABA_A_ [35]	Cell surface	Intermediate	Multifocal encephalitis with non-enhancing white matter MRI lesions	Thymoma
GABA_B_ [18]	Cell surface	Intermediate	Limbic encephalitis	SCLC
NMDAR [123]	Cell surface	Intermediate	Psychiatric prodrome, encephalitis, seizures, hyperkinetic movements, dysautonomia, coma	Teratoma (ovarian or extra-ovarian)
CASPR2 [79–81]	Cell surface	Low-intermediate	Limbic encephalitis, neuromyotonia, Morvan syndrome (cancer risk 50%)	Malignant thymoma

*ANNA* antineuronal nuclear antibody, *Ataxia* cerebellar ataxia or paraneoplastic cerebellar degeneration, *SCLC* small cell lung cancer, *Adeno* adenocarcinoma, *PCA* Purkinje cell antibody, *MAP1B* microtubule-associated protein 1B, *NSCLC* non-small cell lung cancer, *DACH1* Dachshund-homolog 1, *GI* gastrointestinal, *NHL* non-Hodgkin lymphoma, *AGNA* anti-glial nuclear antibody, *SOX1* SRY-box transcription factor 1, *CRMP* collapsin-responsive mediator protein, *LEMS* Lambert-Eaton myasthenic syndrome, *mGluR* metabotropic glutamate receptor, *AMPAR* α-amino-3-hydroxy-5-methyl-4-isoxazolepropionic acid receptor, *GABA*_*A*_ gamma-aminobutyric acid-A receptor, *GABA*_*B*_*R* gamma-aminobutyric acid-B receptor, *NMDAR* N-methyl-d-aspartate receptor, *CASPR2* contactin-associated protein-like 2

**Table 3:** Paraneoplastic antibodies: novel, less common and lower risk

Antibody (alternative name)	Risk	Antigen location	Clinical manifestations	Associated neoplasm type
mGluR2 [124]	High*	Cell surface	Ataxia	SCLC, rhabdomyosarcoma
Zic 4 [125, 126]	High*	Intracellular	Ataxia	SCLC
NF-L [57•]	High*	Intracellular	Ataxia, encephalopathy, myelopathy	Neuroendocrine
PDE10A [127]	High*	Intracellular	Encephalopathy, chorea	Lung, renal, pancreatic
Protein kinase C [128]	High*	Intracellular	Ataxia	NSCLC, hepatobiliary adeno
TRIM 46 [129, 130]	High*	Intracellular	Encephalomyelitis, ataxia	SCLC
TRIM 9/67 [131]	High*	Intracellular	Ataxia	NSCLC (adeno)
CARP VIII [132, 133]	High*	Intracellular	Ataxia	Melanoma, ovarian, breast adenos
BRSK2 [134]	High*	Intracellular	Limbic encephalitis	SCLC
mGluR5 [135]	Intermediate	Cell surface	Encephalitis, Ophelia syndrome	Hodgkin lymphoma, *SCLC*
P/Q VGCC [76]	Intermediate	Cell surface	LEMS, ataxia	SCLC
ARHGAP26 (GRAF1) [136]	Intermediate	Intracellular	Brainstem encephalitis, ataxia	Ovarian adenos
ITPR1 [48]	Intermediate	Intracellular	Brainstem encephalitis, ataxia, peripheral neuropathy	Breast, lung, haematologic
LGI1 [79, 81]	Low	Cell surface	Limbic encephalitis	Malignant thymoma, neuroendocrine
DPPX [37]	Low	Cell surface	Encephalitis, CNS hyperexcitability, chronic diarrhea/weight loss; PERM	B cell neoplasms
GlyR [63, 64]	Low	Cell surface	Limbic encephalitis, PERM	Hodgkin lymphoma, malignant thymoma
Contactin-1 [137, 138]	Low	Cell surface	CIDP (sensory-predominant)	Thymoma, breast adeno, plasmacytoma
Neurochondrin [139]	Low	Cell surface	Ataxia	Uterine adeno
MOG [43]	Low	Cell surface	MOGAD	Ovarian teratoma
Aquaporin 4 [60]	Low	Cell surface	NMOSD	Adenos
α3-AChR [73]	Low	Cell surface	Autonomic neuropathy, gastrointestinal pseudo-obstruction	Adenos
GFAP [4•, 38]	Low	Intracellular	Meningoencephalitis, optic disc oedema	Ovarian teratoma, various adenos
GAD65 [62]	Low	Intracellular	Limbic encephalitis, stiff-person syndrome, ataxia	SCLC, other neuroendocrine, malignant thymoma
Adaptor protein 3B2 [140]	Low	Intracellular	Ataxia, sensory ataxia	Renal carcinoma, B cell lymphoma
Septin 7 [51]	Low	Cell surface	Encephalopathy, myelopathy, psychiatric symptoms	Breast adenos, NHL

*mGluR* metabotropic glutamate receptor, *Ataxia* cerebellar ataxia or paraneoplastic cerebellar degeneration, *SCLC* small cell lung cancer, *NF-L* neuronal intermediate filament, *PDE10A* phosphodiesterase 10A, *NSCLC* non-small cell lung cancer, *Adeno* adenocarcinoma, *TRIM* tripartite motif-containing protein, *CARP VIII* carbonic anhydrase-related protein VIII, *BRSK2* BR serine/threonine kinase 2, *P/Q VGCC* P/Q type voltage-gated calcium channel, *LEMS* Lambert Eaton myasthenic syndrome, *ARHGAP26* Rho GTPase-activating protein, *GRAF1* GTPase regulator associated with focal adhesion kinase 1, *ITPR1* inositol 1,4,5-triphosphate receptor 1, *LGI1* leucine-rich glioma inactivated protein 1, *DPPX* dipeptidyl-peptidase-like protein, *CNS* central nervous system, *PERM* progressive encephalomyelitis with rigidity and myoclonus, *GlyR* glycine receptor, *CIDP* chronic inflammatory demyelinating polyneuropathy, *MOG* myelin oligodendrocyte glycoprotein, *MOGAD* myelin oligodendrocyte glycoprotein antibody disease, *NMOSD* neuromyelitis optic spectrum disorder, *α3-AChR* ganglionic nicotinic acetylcholine receptor, *GFAP* glial fibrillary acidic protein, *GAD* glutamic acid decarboxylase
